# Fossil leaf wax hydrogen isotopes reveal variability of Atlantic and Mediterranean climate forcing on the southeast Iberian Peninsula between 6000 to 3000 cal. BP

**DOI:** 10.1371/journal.pone.0243662

**Published:** 2020-12-23

**Authors:** Julien Schirrmacher, Nils Andersen, Ralph R. Schneider, Mara Weinelt

**Affiliations:** 1 CRC1266, Christian-Albrechts University, Kiel, Germany; 2 Leibniz Laboratory for Radiometric Dating and Stable Isotope Research, Christian-Albrechts University, Kiel, Germany; 3 Institute of Geosciences, Christian-Albrechts University, Kiel, Germany; University at Buffalo - The State University of New York, UNITED STATES

## Abstract

Many recently published papers have investigated the spatial and temporal manifestation of the 4.2 ka BP climate event at regional and global scales. However, questions with regard to the potential drivers of the associated climate change remain open. Here, we investigate the interaction between Atlantic and Mediterranean climate forcing on the south-eastern Iberian Peninsula during the mid- to late Holocene using compound-specific hydrogen isotopes from fossil leaf waxes preserved in marine sediments. Variability of hydrogen isotope values in the study area is primarily related to changes in the precipitation source and indicates three phases of increased Mediterranean sourced precipitation from 5450 to 5350 cal. BP, from 5150 to 4300 cal. BP including a short-term interruption around 4800 cal. BP, and from 3400 to 3000 cal. BP interrupted around 3200 cal. BP. These phases are in good agreement with times of prevailing positive modes of the North Atlantic Oscillation (NAO) and reduced storm activity in the Western Mediterranean suggesting that the NAO was the dominant modulator of relative variability in precipitation sources. However, as previously suggested other modes such as the Western Mediterranean Oscillation (WeMO) may have altered this overall relationship. In this regard, a decrease in Mediterranean moisture source coincident with a rapid reduction in warm season precipitation during the 4.2 ka BP event at the south-eastern Iberian Peninsula might have been related to negative WeMO conditions.

## Introduction

In recent years much effort has been made in reconstructing and understanding the socio-environmental dynamics associated with the 4.2 ka BP event. Initially, the 4.2 ka BP event was described as an “archaeological event” in the Near East, where the Akkadian Empire potentially collapsed due to an increase in regional aridity [1]. Similar climatic related collapses or transformations within ancient societies at that time have also been documented in different regions across the northern hemisphere [2–4] including southern Iberia [5, 6]. However, regional heterogeneity in both, climatic conditions and social developments, have been indicated within the Mediterranean region [6–8]. Still, the narrative of a megadrought affecting ancient societies across Asia, the Mediterranean, and northeast Africa arose [9, 10].

Such a first evidence of climate related collapses or transformations in ancient societies have promoted intense investigations of the “climatic” 4.2 ka BP event, which resulted in a variety of associated paleoclimatic studies from the Mediterranean area [6, 7, 11, 12], Asia [13–15], North America [16], the northern North Atlantic region [17–19], and the southern hemisphere as well [20, 21]. Altogether, paleoclimatic studies point to a series of climatic anomalies between 4400 and 3800 cal. BP, which across the Mediterranean region are often registered as dry and cool events [6, 7]. But, climatic conditions may have been variable on a regional and seasonal scale with humid conditions prevailing occasionally [6, 11, 22].

However, potential drivers of the more widespread drier and cooler climate periods across the mid-latitudes of the northern hemisphere are not yet understood. Since the North Atlantic Oscillation (NAO) is modulating winter precipitation across large parts of the Mediterranean region, in particular the Western Mediterranean [23, 24], it is often regarded as one important driver for drought associated with the 4.2 ka BP event [11, 25, 26]. At that time, a major NAO-like forcing is also suggested by modelling studies [27]. On the other hand, recent studies have indicated that the 4.2 ka BP event in the Mediterranean region could have been more pronounced during the summer season [6, 7, 28]. Thus, the search for a potential driver of the climatic 4.2 ka BP event particularly in the Western Mediterranean region has to include seasonal variability with Atlantic winter versus Mediterranean summer forcing.

**Fig 1:**
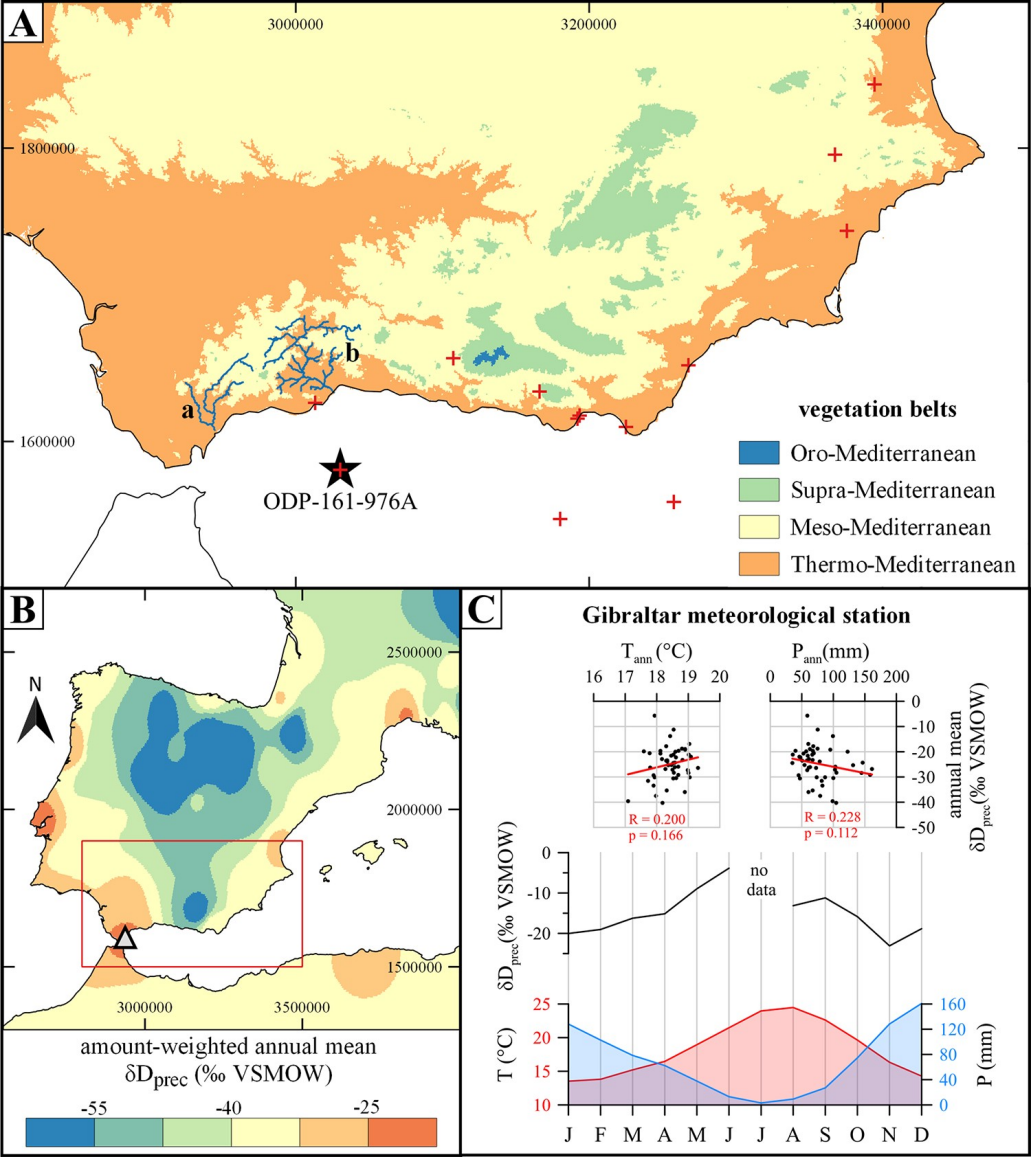
Study area. A) Major vegetation belts at the south-eastern Iberian Peninsula are shown along with main rivers Guadiaro (a) and Guadalhorce (b) in the study area. Vegetation belts have been calculated after *[29]* based on 0.5° gridded climate data from WorldClim V2.0 *[30]*. Star indicates the location of marine sediment core ODP-161-976A and red crosses show locations of additional archives used for regional paleoclimatological analysis (see Table 1 for more detailed information): Navarrés *[31]*, Villena *[32]*, Elx *[33]*, Antas *[34]*, Padul *[26]*, Sierra de Gádor *[35]*, Roquetas de Mar *[34]*, San Rafael *[34]*, Cabo de Gata *[36]*, El Refugio *[37]*, TTR14-300G *[38]*, ODP-161-976A *[11, 39]* and, MD95-2043 *[7, 40–42]*. B) Map showing the spatial distribution of amount-weighted long-term (1961–2016) annual mean hydrogen isotopic composition of precipitation (δD_prec_). The raw data was downloaded from the Global Network of Isotopes in Precipitation (GNIP) database and interpolated using an inverse distance weighted (IDW) approach (unlimited search radius and power value = 3.0). Red square indicates area shown in A). Triangle denotes the location of Gibraltar meteorological station, which data is shown to the right. C) Average monthly air temperature (T), precipitation (P) and, amount-weighted δD_prec_ of Gibraltar meteorological station for the period 1961–2016 (bottom) as well as the correlation of their annual means (top). All raw data from Gibraltar meteorological station have been downloaded from the GNIP database. Please note, that no δD_prec_ data is available for July due to the scarce precipitation during that month.

To shed further light on the potential driver of climate variability around 4200 cal. BP, we investigated the temporal variability of the interaction between Atlantic and Mediterranean climatic regimes. Therefore, we analysed compound-specific hydrogen and carbon isotopes from fossil leaf waxes, i.e. land-derived *n*-alkanes, as tracer of past atmospheric circulation patterns in a well-suited marine sediment core from the Alboran Sea–an area actually located at the interface of Atlantic and Mediterranean climate regimes (Fig 1).

### Study area

The terrestrial organic compounds investigated in this study are mainly sourced from the catchment areas of the Guadiaro and Guadalhorce rivers (Fig 1). Both catchment areas are draining the Thermo- and Mesomediterranean vegetation belts (Fig 1). These vegetation belts in the southern Iberian Peninsula are generally characterized by scrubland and grassland with only patchy forests in favourable locations such as water courses [29]. While the main tree species are different *Quercus* species, water courses are dominated by *Salix, Fraxinus*, and *Ulmus* [29]. Important shrub species in the study area are *Juniperus, Phillyrea, Erica, Olea*, and *Arbutus unedo* [29].

Most of the aforementioned species are adapted to the high seasonality in the study area, which is currently under a Mediterranean climate influence characterized by a cool and rainy winter season and a hot and dry summer season. Based on data from the Spanish State Meteorological Agency (AEMET) modern (1981–2010) winter conditions in the study area range between a minimum air temperature of 12.1°C at Málaga airport and 13.0°C at Tarifa close to Gibraltar. At these stations, precipitation reveals a maximum during winter of up to 100 and 118 mm at Málaga and Tarifa stations, respectively. In contrast, summer conditions are characterized by maximum temperatures between 22.3 and 26.0°C at Tarifa and Málaga stations. Between June and August there is almost no precipitation occurring at both stations.

**Fig 2:**
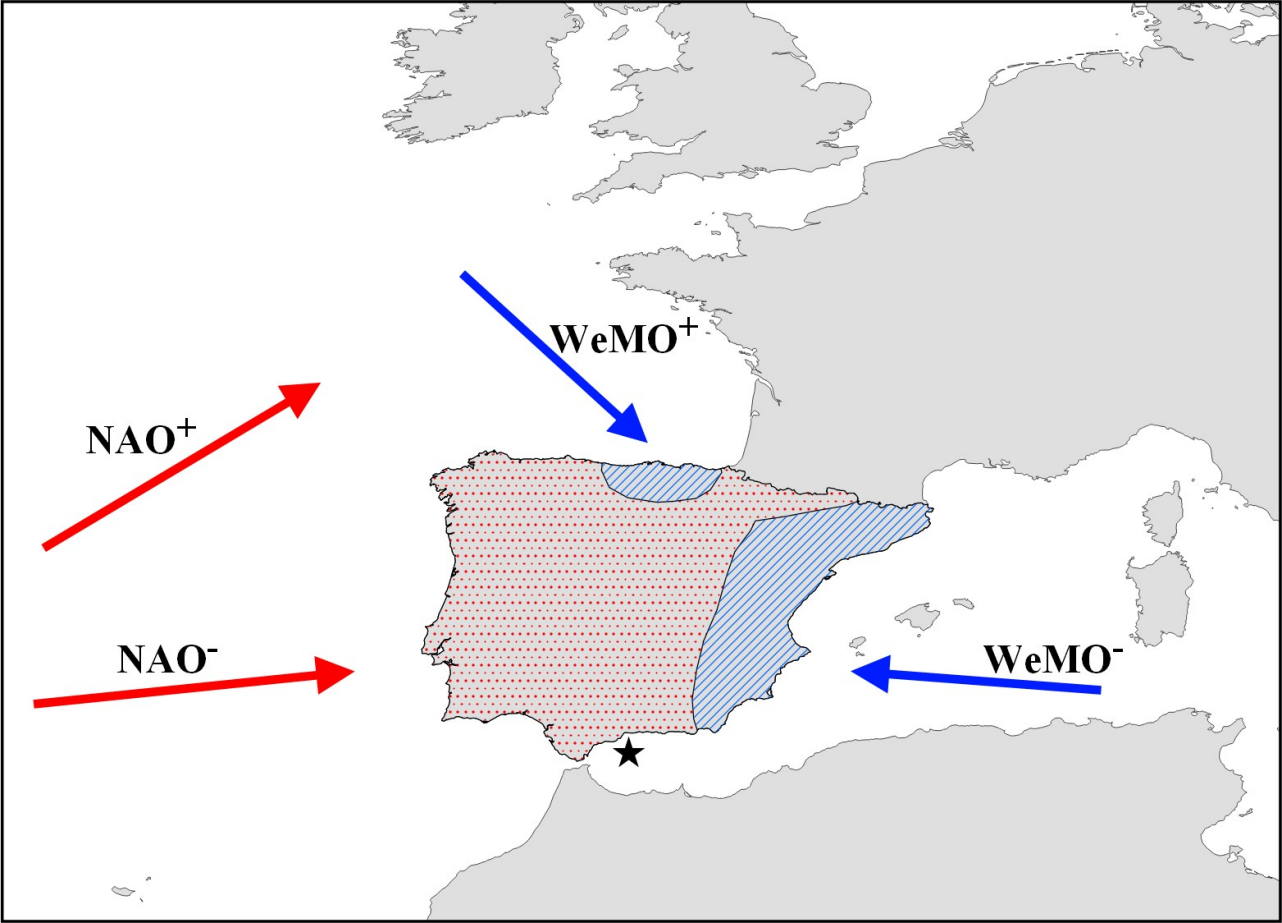
Main atmospheric drivers of temporal precipitation variability on the Iberian Peninsula. Map showing areas on the Iberian Peninsula, where temporal winter (October–March) precipitation variability is dominated by the NAO (dotted red) and the WeMO (hatched blue). Areas have been redrawn from *[45]*. Red arrows indicate dominant wind direction under NAO^+^ and NAO^-^ conditions, respectively. Blue arrows indicate the same for WeMO^+^ and WeMO^-^ conditions. Star indicates the location of marine sediment core ODP-161-976A.

The temporal variability of precipitation in the study area is mainly controlled by the North Atlantic Oscillation (NAO), which primarily transports moisture from the Atlantic during the winter season [24, 43, 44]. During positive NAO (NAO^+^; i.e. a high difference in sea level pressure between the Azores and Iceland) the study area experiences drier conditions, because the main storm track of the westerlies lead towards northern and central Europe (Fig 2), and *vice versa*. A secondary mode responsible for temporal climate variability in the area is the Western Mediterranean Oscillation (WeMO) [45]. In its positive phase (WeMO^+^) the Western Mediterranean Oscillation is associated with relatively cool and dry north-westerly winds, while during its negative phase (WeMO^-^) relatively warm and humid easterly winds are prevailing (Fig 2). The WeMO is active throughout the year, but WeMO^-^ conditions are particularly associated with increased winter precipitation along the Catalonian and Valencian coasts [45, 46].

The scarce precipitation during the summer season in the study area, when the NAO driven influence is less pronounced [47], is mainly driven by mesoscale synoptic patterns and local convective systems [48]. These are responsible for short torrential rainfall events, which are particularly evident along the Mediterranean coast during times of a high land-sea temperature contrast [48]. Such a high land-sea temperature contrast may be promoted by the advection of cool northerly air masses under WeMO^+^ conditions during summer [46].

Overall, both atmospheric modes–the NAO and the WeMO–modulate the temporal variability of the two main precipitation sources at the Iberian Peninsula, which are an Atlantic source dominant during winter and a Mediterranean source during summer [49]. These two precipitation sources are also reflected in the spatial distribution of the hydrogen isotopic composition within the amount-weighted long-term annual mean precipitation (δD_prec_). In the Atlantic dominated inland areas amount-weighted annual mean δD_prec_ values are typically below -40 ‰ VSMOW, while coastal areas, which are more intensely affected by local precipitation sources, vary between -15 and -35 ‰ VSMOW (Fig 1). Moreover, at Gibraltar meteorological station amount-weighted δD_prec_ values show no significant correlation with precipitation amount or temperature on the annual scale (Fig 1). However, on the monthly scale amount-weighted δD_prec_ values are highly correlated with precipitation amount (R = 0.849; p < 0.001) and temperature (R = 0.756; p = 0.004) (not shown). This is because the precipitation source, which generally varies on a seasonal scale (Atlantic winter and Mediterranean summer) [49], might be responsible for the observed seasonal variability of amount-weighted δD_prec_ values in the study area [48].

### *n*-Alkane data as paleoclimatic proxy

*n*-Alkanes are synthesized by terrestrial plants as leaf waxes in order to protect themselves against water loss due to evapotranspiration [50]. After eolian or riverine transport, leaf waxes are deposited in soils, lakes, and marine sediments [51–53]. After deposition in the sediments, *n*-alkanes are relatively resistant to diagenesis [54, 55] and may serve as paleoclimatic indicator. Potential alteration of *n*-alkanes can be investigated by the carbon preference index (CPI), which illustrates the ratio of odd versus even chain lengths [56]. Usually, a CPI above 2 indicates that *n*-alkanes are not altered and, thus, can be reliably used as paleoclimatic indicator [57]. The average chain length (ACL) of *n*-alkanes might be used as such a paleoclimatic indicator since it has been found to record regional aridity in Mediterranean settings [58, 59]. This is based on the observation, that plants produce on average longer *n*-alkane homologues resulting in an increasing ACL, when water availability is reduced [60]. Also, isotopic analyses of individual *n*-alkanes, have been used extensively during the last years in tropical [61–64] but also in Mediterranean regions [65–68] in order to assess terrestrial environmental and climatic parameters.

Analysing individual *n*-alkane homologues for carbon isotopes (δ^13^C_Cx_) provides important information on the distribution of C3 and C4 plants [69]. Due to their different photosynthetic pathway, C4 plants typically exhibit elevated δ^13^C_Cx_ values varying between -20 to -15 ‰ compared to those from C3 plants, which vary between -45 to -30 ‰ [69, 70]. In environmental settings, which are characterized by stability of the vegetational record with regard to C3 vs. C4 plant distribution, δ^13^C_Cx_ values may also record plant water stress [71]. Increasing δ^13^C_Cx_ values would point to isotopic enrichment within the plant’s source water pool due to enhanced evapotranspiration. In Mediterranean settings, δ^13^C_Cx_ data of the long chain *n*-alkanes have been suggested to be most suitable for studying changes in humidity [58].

In settings mixed with C3 and C4 plants, the δ^13^C_Cx_ values are further needed for potential correction of the hydrogen isotopic data of the individual *n*-alkanes (δD_Cx_) [72, 73]. Despite of potential alteration through the vegetation type, the δD_Cx_ data is highly correlated with that of the water source, i.e. precipitation (δD_prec_) during the plants growing season [74]. Additional factors controlling δD_prec_ and thus, also δD_Cx_ are atmospheric temperature, the amount of rainfall, evapotranspiration, and precipitation source [74–76].

## Materials and methods

### Sediment core and age model

Sediment core ODP-161-976A (36°12.320’ N; 4°18.760’ W; 1108m water depth) was retrieved in the Alboran Sea during the JOIDES RESOLUTION cruise in 1995 [77]. The sampling of this sediment core was already described in a previous paper [11]. To achieve multi-decadal resolution, the section from 100.0 to 149.0 cm was continuously sampled at 0.5 cm distances in the IODP (International Ocean Discovery Program) core repository at MARUM (Center for Marine Environmental Sciences) in Bremen (Germany). Also, the age model of sediment core ODP-161-976A has already been published in earlier publications [6, 11]. The final age model of ODP-161-976A is based on 11 AMS ^14^C dates. The sediment core encompasses an analysed time period between ca. 5750 to 3000 cal. BP with a temporal resolution varying between 8 and 114 years for ODP-161-976A.

### Sample preparation and calculations

The sample preparation of sediment core ODP-161-976A followed the protocol of the biomarker laboratory at Kiel University and has already been described in an earlier study [11]. In short, *n*-alkanes were extracted from the freeze-dried and finely ground sediment samples with an accelerated solvent extractor (ASE-200, Dionex) at 100 bar and 100°C using a 9:1 (v = v) mixture of dichloromethane (DCM) and methanol. After extraction samples were de-sulfured by stirring for 30 minutes with activated copper. The de-sulfured *n*-alkanes were subsequently separated by silica gel column chromatography using activated silica gel and hexane. *n*-Alkanes were further separated using silver nitrate (AgNO_3_) coated silica gel. Subsequently, individual *n*-alkane homologues have been identified with an Agilent 6890N gas chromatograph equipped with a Restek XTI-5 capillary column (30 m x 320 μm x 0.25 μm) based on the comparison of their retention times with an external standard containing a series of *n*-alkane homologues of known concentration. On this basis, *n*-alkanes were also quantified using the FID peak areas calibrated against the external standard. The concentrations of odd terrestrial *n*-alkanes are provided in the supplement of this article.

Based on the quantified *n*-alkane concentrations the carbon preference index (CPI) has been calculated in order to assess potential alteration of the sedimentary *n*-alkanes and thus, their reliability as paleoclimatic indicator:

$${\mathrm{CPI}}_{27‐33}=0.5\;*\;\left(\frac{\left({n‐C}_{27}+{n‐C}_{29}+{n‐C}_{31}+{n‐C}_{33}\right)}{\left({n‐C}_{26}+{n‐C}_{28}+{n‐C}_{30}+{n‐C}_{32}\right)}+\frac{\left({n‐C}_{27}+{n‐C}_{29}+{n‐C}_{31}+{n‐C}_{33}\right)}{\left({n‐C}_{28}+{n‐C}_{30}+{n‐C}_{32}+{n‐C}_{34}\right)}\right)$$ (1)

As paleoclimatic indicator the average chain length (ACL) has been calculated following Norström et al. [58]:

$${\mathrm{ACL}}_{29‐35}=\frac{{29\;*\;n‐C}_{29}+{31\;*\;n‐C}_{31}+{33\;*\;n‐C}_{33}+{35\;*\;n‐C}_{35}}{{n‐C}_{29}+{n‐C}_{31}+{n‐C}_{33}+{n‐C}_{35}}$$ (2)

In both equations *n*-C_x_ refers to the concentration of the *n*-alkane with x carbon atoms.

### Compound-specific isotope analysis

For this study, terrestrial-sourced *n*-alkane homologues of sufficient concentration (i.e. *n*-C_29_, *n*-C_31_, and *n*-C_33_) have been analysed by gas chromatography-isotope ratio mass spectrometry (GC-IRMS) for δD and δ^13^C at the Leibniz Laboratory for Radiometric Dating and Stable Isotope Research at Kiel University. Samples have been measured on an Agilent 6890 gas chromatograph equipped with a Gerstel KAS 4 PTV injector and an Agilent DB-5 capillary column (30 m x 250 μm x 0.25 μm) coupled to a Thermo Scientific MAT 253 isotope ratio mass spectrometer (IRMS). Depending on the *n*-alkane concentration, between 5 and 30 μl of each sample has been injected 2–4 times in order to achieve a statistically robust analytical error for each *n*-alkane homologue. The δD and δ^13^C values are reported relative to Vienna Standard Mean Ocean Water (‰ VSMOW) based on Arndt Schimmelmann’s A6 reference mixture from 2015 and Vienna Pee Dee Belemnite (‰ VPDB) scales using Arndt Schimmelmann’s A7 reference mixture from 2017, respectively.

For final evaluation of the δD and δ^13^C data, their weighted-mean averages (WMAs) of all three individual isotopic records have been calculated according to the following equation:

$${\delta D}_{\mathrm{WMA}}=\sum {\delta D}_{x}*(\frac{{n‐C}_{x}}{\left({n‐C}_{29}+{n‐C}_{31}+{n‐C}_{33}\right)})$$ (3)

with δD_x_ and *n*-C_x_ representing the hydrogen isotopic value and the concentration of the *n*-alkane with x carbon atoms, respectively. The same equation has been applied to calculate the δ^13^C_WMA_ data.

### Regional analysis

The regional analysis of past seasonal precipitation development is based on a compilation of various climatic proxies from speleothems, marine, lacustrine, and terrestrial archives from the Iberian Peninsula published in a previous publication [6]. For this study, this compilation has been regionally subsampled for the Thermo- and Mesomediterranean vegetation belts in the southeast of the Iberian Peninsula providing new analysis on a regional scale. Furthermore, the ACL_29-35_ calculated in this study has been included into the compilation. Altogether, 14 records are reflecting annual, 11 records are reflecting cold season, and 2 records are reflecting warm season precipitation variability (Table 1; Fig 1). The interpretational background of the used proxies is explained in detail in a previous publication [6] or in the individual publications listed in Table 1. However, because the majority of archives are based on pollen percentages, in the following their interpretational background is briefly recalled. Based on the modern relationship between arboreal pollen and cold season precipitation on the Iberian Peninsula [78], we interpreted decreasing arboreal pollen percentages as indicator for decreasing cold season precipitation. This is further in line with the application in other paleoclimatological studies from the area [26, 40]. On the other hand, xerophytic species including Chenopodiaceae, Amaranthaceae, and *Artemisia* have been shown to be indicative of prolonged annual dry periods [79]. Consequently, an increase in xerophytic pollen percentages have been interpreted as indication of dry annual conditions.

**Table 1:** Dataset used for the regional precipitation analysis.

Site	Season	Archive	Proxy	Reference
Antas	annual	terrestrial	Xerophytes	[34]
El Refugio Cave	annual	speleothem	Stalagmite density	[37]
Elx	annual	terrestrial	Xerophytes	[33]
MD95-2043	annual	marine	MAT on pollen	[7, 40–42]
MD95-2043	annual	marine	Xerophytes	[41]
Navarrés	annual	terrestrial	WAPLS on pollen	[31]
ODP-161-976A	annual	marine	MAT on pollen	[6,39]
Padul	annual	terrestrial	Xerophytes	[26]
Roquetas de Mar	annual	terrestrial	Xerophytes	[34]
San Rafael	annual	terrestrial	Xerophytes	[34]
San Rafael	annual	terrestrial	WAPLS on pollen	[31]
Sierra de Gádor	annual	lake	Xerophytes	[35]
TTR14-300G	annual	marine	La/Lu ratio	[38]
ODP-161-976A	annual	marine	ACL_29-35_	this study
Antas	cold season	terrestrial	arboreal pollen	[34]
Cabo de Gata	cold season	terrestrial	arboreal pollen	[36]
Elx	cold season	terrestrial	arboreal pollen	[33]
MD95-2043	cold season	marine	MAT on pollen	[7, 40–42]
MD95-2043	cold season	marine	arboreal pollen	[41]
ODP-161-976A	cold season	marine	MAT on pollen	[6,39]
Padul	cold season	terrestrial	arboreal pollen	[26]
Roquetas de Mar	cold season	terrestrial	arboreal pollen	[34]
San Rafael	cold season	terrestrial	arboreal pollen	[34]
Sierra de Gádor	cold season	lake	arboreal pollen	[35]
Villena	cold season	terrestrial	pollen ratio	[32]
MD95-2043	warm season	marine	MAT on pollen	[7, 40–42]
ODP-161-976A	warm season	marine	MAT on pollen	[6, 39]

MAT = modern analogue technique, WAPLS = weighted-average partial least squares regression technique.

The z-scores [80] of all paleoclimatic proxies reflecting either annual, cold, or warm season precipitation have been combined to regional time-series of qualitative precipitation change. Prior to analysis, the speleothem data of El Refugio Cave [37], which has an average temporal resolution of 3 years, has been downscaled to a temporal resolution of 50 years. The calculation of 50-year means prevents the over-representation of this archive in the regional time-series since it had a much higher temporal resolution compared to all other archives. The regional time-series have then been smoothed using a gaussian LOESS smooth with a 2^nd^ order polynomial and a smoothing parameter of 0.2.

## Results

**Fig 3:**
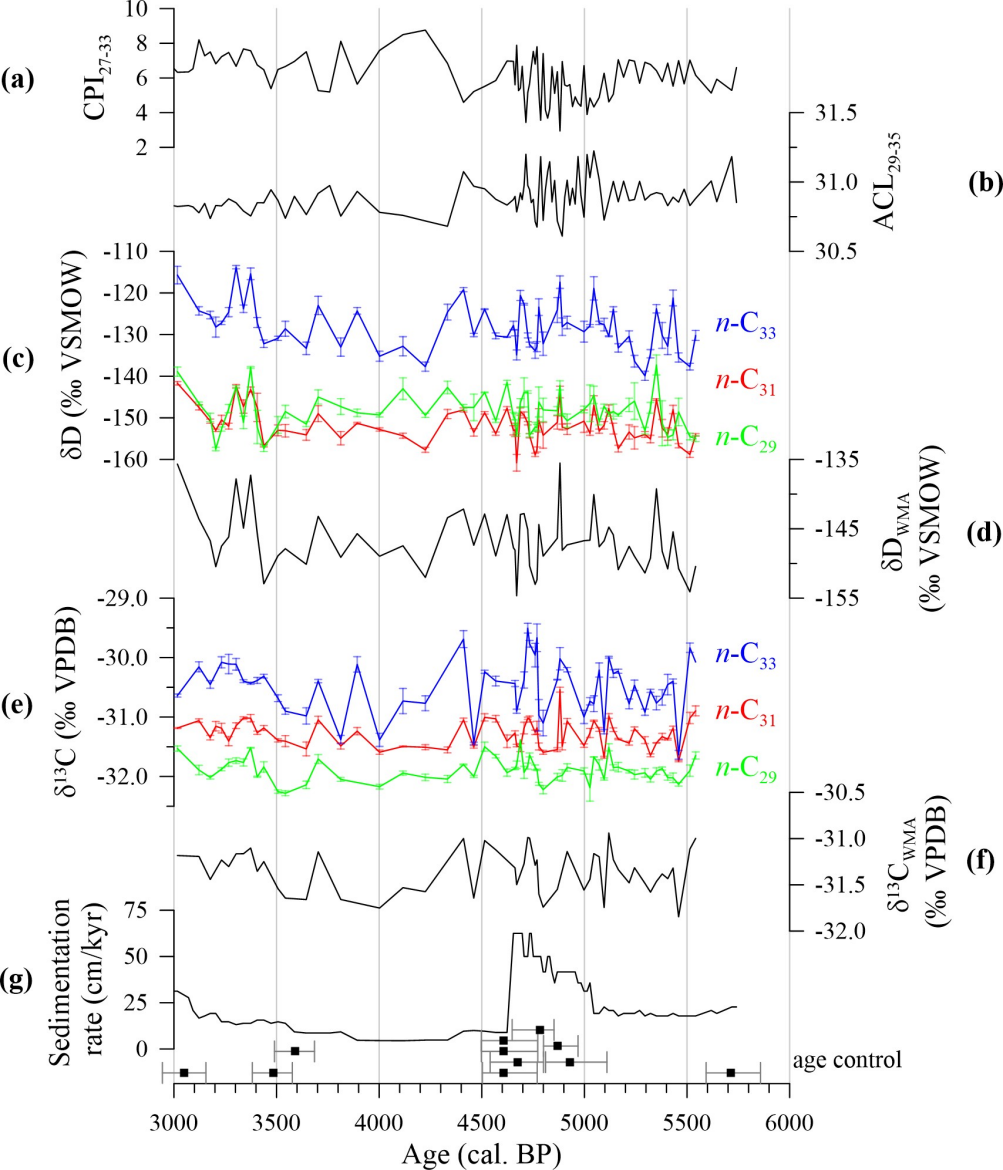
Molecular and isotopic n-alkane data from ODP-161-976A. a) CPI_27-33_, b) ACL_29-35_, c) hydrogen isotopic data of individual n-alkane homologues δD_C33_ (blue), δD_C31_ (red), and δD_C29_ (green), d) weighted-mean average of all individual hydrogen isotopic records (δD_WMA_), e) carbon isotopic data of individual n-alkane homologues δ^13^C_C33_ (blue), δ^13^C_C31_ (red), and δ^13^C_C29_ (green), f) weighted-mean average of all individual carbon isotopic records (δ^13^C_WMA_), and g) sedimentation rate. Black squares show calibrated AMS ^14^C dates and their according uncertainties.

The carbon preference index for odd *n*-alkanes between 27 and 33 carbon atoms (CPI_27-33_) varies around mean of 6.1 with a minimum of 2.9 and a maximum of 8.7 (Fig 3). The average chain length (ACL) has been calculated for odd *n*-alkanes with carbon atoms ranging between 29 and 35. The ACL_29-35_ reveals no long-term trend and varies between 31.2 and 30.6 with a mean of 30.9 (Fig 3).

Carbon and hydrogen isotopic data of three *n*-alkane homologues (*n*-C_29_, *n*-C_31_, and *n*-C_33_) are presented for the period between ca. 5800 and 3000 cal. BP. In this interval, the carbon isotopic values of the *n*-C_29_ homologue (δ^13^C_C29_) vary between -31.4 and -32.3 ‰ (Fig 3). δ^13^C_C31_ values vary between -30.5 and -31.7 ‰, while δ^13^C_C33_ values range from -29.5 to -31.6 ‰. There is no obvious trend in any of the carbon isotopic time series, but average values progressively increase with increasing chain length. δ^13^C_C29_ values exhibit an average of -31.9 ‰, while δ^13^C_C31_ values vary around a mean of -31.3 ‰ and δ^13^C_C33_ values are on average -30.5 ‰ (Fig 3). The weighted-mean average of the carbon isotopes δ^13^C_WMA_ varies between -31.8 and -30.9 ‰ with a mean value of -31.3 ‰ (Fig 3).

**Fig 4:**
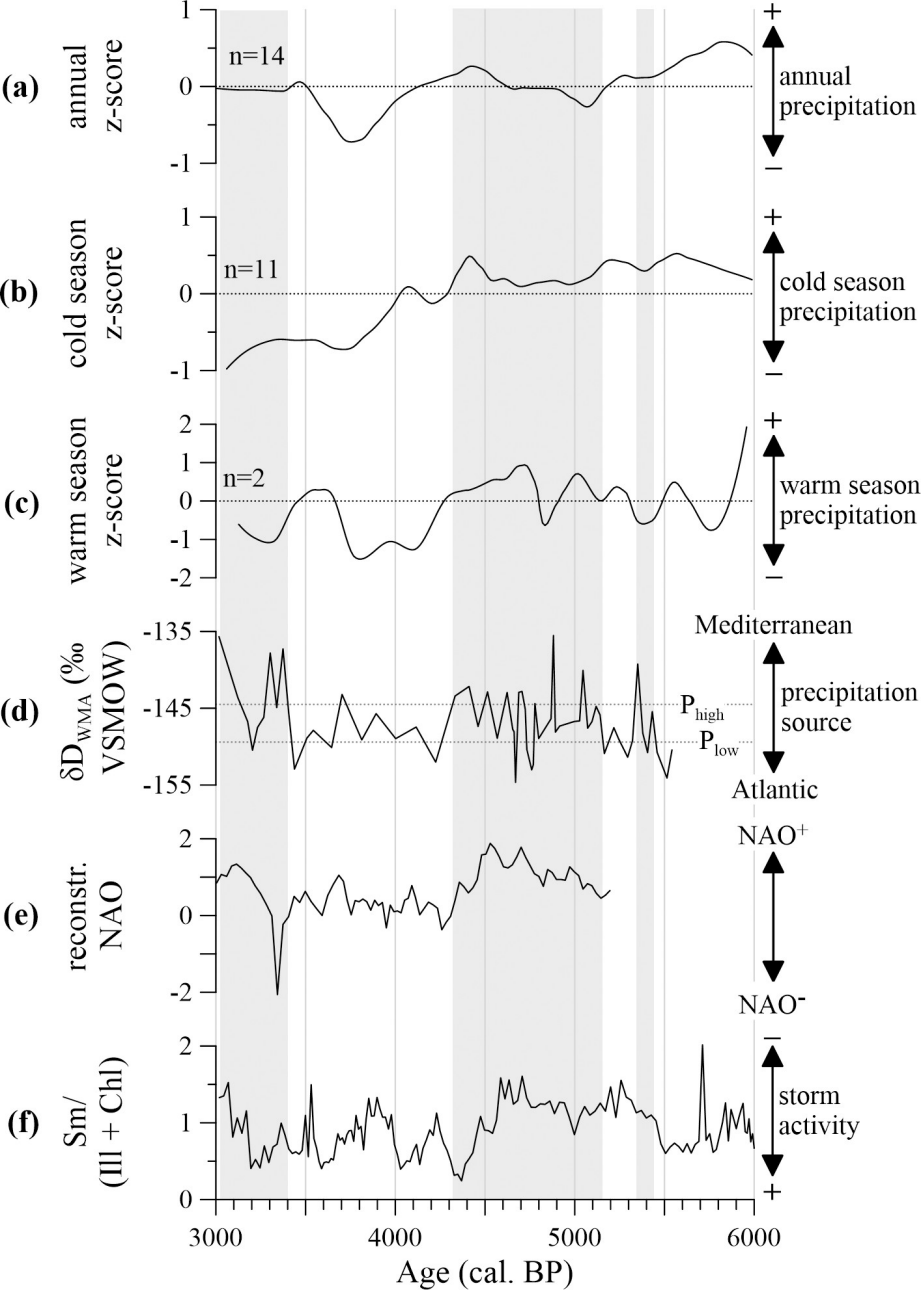
Atlantic versus Mediterranean influence. a) Regional z-score of annual precipitation variability, b) regional z-score of cold season precipitation variability, and c) regional z-score of warm season precipitation variability. Numbers of individual archives (n) included into the regional data are also shown. d) Weighted-mean average hydrogen isotopic data (δD_WMA_) is displayed together with means of periods characterized by prolonged high (P_high_) and low δD_WMA_ values (P_low_) for orientation (see results-section). e) Reconstructed NAO from lake SS1200 in Greenland *[87]* and f) clay mineral ratio of Smectite (Sm), Illite (Ill), and Chlorite (Chl) from Gulf of Lions sediment core PB06 indicative of Western Mediterranean storminess *[88]* are shown for interpretation. Vertical grey bars indicate periods of increased Mediterranean precipitation in southeast Iberia based on the hydrogen isotopic data from ODP-161-976A.

Within the studied timespan, the hydrogen isotopic values vary between -137.3 ‰ and -157.5 ‰ for the *n*-C_29_ homologue (δD_C29_), between -141.7 ‰ and -160.8 ‰ for δD_C31_, and between -113.8 ‰ and -139.9 ‰ for δD_C33_ (Fig 3). While the absolute values and the amplitude of δD_C29_ and δD_C31_ are similar, δD_C33_ values are slightly higher and variability is of larger amplitude. Apart from these differences, hydrogen isotopic values of all three homologues reveal a slightly increasing trend and significantly covary in the analysed period (Table 2). Based on the weighted-mean combination of all three δD records, periods of high and low isotopic values can be distinguished in the δD_WMA_ data (Fig 4). High δD_WMA_ values (-144.5 ‰ on average) are evident around 5400, from 5100 to 4300 with a short-term decrease around ca. 4800 cal. BP, from 3400 to 3300, and at 3000 cal. BP. In contrast, low δD_WMA_ values averaging -149.4 ‰ are noticed at 5500, at 5300, and from 4200 to 3400 cal. BP.

**Table 2:** Correlation of carbon and hydrogen isotopic data from ODP-161-976A.

	δ^13^C_C31_	δ^13^C_C33_		δD_C31_	δD_C33_
**δ**^**13**^**C**_**C29**_	R = 0.622	R = 0.540	**δD**_**C29**_	R = 0.856	R = 0.534
	p < 0.001	p < 0.001		p < 0.001	p < 0.001
**δ**^**13**^**C**_**C31**_		R = 0.733	**δD**_**C31**_		R = 0.636
		p < 0.001			p < 0.001

## Discussion

### Drivers of hydrogen isotopic variability

Variability of hydrogen isotopic data from individual *n*-alkane homologues is potentially driven by five major parameters, which are changes in (1) vegetation types, (2) precipitation amount, (3) atmospheric temperature, (4) evapotranspiration, and (5) precipitation source [73–75]. In the following, we conclude that the variability of hydrogen isotopic values from individual *n*-alkanes originating from southeast Iberia and deposited in marine sediment core ODP-161-976A is primarily driven by changes in the source of precipitation.

Several studies have highlighted the dependence of *n*-alkane hydrogen isotopic composition on the distribution of C3 and C4 plants in modern [81] and paleoclimatic studies [72, 82, 83]. This is due to the different photosynthetic regulatory pathways of these plant types, which results in a different apparent fractionation between the hydrogen isotopic value of precipitation (δD_prec_) and that of *n*-alkanes (δD_Cx_) [73]. Any variation in the C3 vs. C4 plant distribution can be tested in parallel through *n*-alkane δ^13^C values. Typically, C4 plants exhibit δ^13^C_Cx_ values between -20 to -15 ‰, while δ^13^C_Cx_ values of C3 plants vary between -45 to -30 ‰ [69, 70]. In our case, the similarity and low variability in absolute values for all three compound-specific carbon isotopic data (δ^13^C_C29_, δ^13^C_C31_, and δ^13^C_C33_) in sediment core ODP-161-976A suggest a dominant C3 vegetation cover throughout the entire period (Fig 3). This is in line with various pollen records from southeast Iberia, which show a dominance of C3 vegetation and no major change towards increased C4 vegetation between 6000 and 3000 cal. BP [26, 39, 41]. Furthermore, δ^13^C and δD values within each *n*-alkane homologue, including the weighted-mean average (WMA) isotopic records, are not correlated at a significant level, with the exception of the *n*-C_31_ homologue revealing a significant but moderate correlation (Table 3). This rules out any significant changes in plant types that may have affected the hydrogen isotopic data at the core site.

**Table 3:** Correlation of paleoclimatological parameters from ODP-161-976A.

	δD_C29_	δD_C31_	δD_C33_	δD_WMA_
***n*-C**_**x**_ **conc.**	R = 0.045	R = 0.084	R = 0.367	
	p = 0.725	p = 0.529	p = 0.004	
**δ**^**13**^**C**_**x**_	R = 0.176	R = 0.363	R = 0.245	R = 0.310
	p = 0.196	p = 0.005	p = 0.073	p = 0.025
**ACL**_**29-35**_	R = 0.167	R = 0.281	R = 0.351	R = 0.377
	p = 0.206	p = 0.033	p = 0.007	p = 0.004
**alkenone SST**	R = 0.184	R = 0.114	R = 0.100	R = 0.195
	p = 0.160	p = 0.382	p = 0.440	p = 0.138

Correlation indices are given for the hydrogen isotopes of every individual *n*-alkane and their carbon isotopic values, their individual concentrations, cumulative concentration of long-chained odd *n*-alkanes, and the alkenone-based SST. Cross-plots of individual parameters are provided in the supplement of this article.

Other crucial parameters driving the hydrogen isotopic signal of *n*-alkanes are changes in atmospheric temperature, precipitation amount, and related evapotranspiration [73, 74, 84]. Meteorological data from Gibraltar implies a general connection of δD_prec_ values to modern changes in the amount of precipitation and atmospheric temperature on a monthly scale (Fig 1). However, no significant statistical correlation of δD_prec_ values with precipitation amount (R = 0.228, p = 0.112) and atmospheric temperature (R = 0.200, p = 0.166) exists during modern times on the annual scale (Fig 1). Moreover, there is also no significant correlation between all three compound-specific hydrogen isotope records and their concentration as well as with other ODP-161-976A data such as the ACL_29-35_ (reflecting changes in regional aridity [58]) and alkenone-based annual mean SST (Table 3), which are closely coupled to atmospheric temperature variability in southeast Iberia [6]. However, correlations between the δD_Cx_ records appear to increase with chain length (Table 3). Overall, a moderate correlation of ACL_29-35_ and the δD_WMA_ record suggests that regional aridity may have had a minor influence on the hydrogen isotopic records. Aridity is closely related to evapotranspiration, which is potentially an important parameter to be considered, when interpreting δD_Cx_ data in semi-arid climates such as southeast Iberia. Evapotranspiration can be studied using δ^13^C values, which are claimed to record plant water stress [71, 85]. As noted before, all individual δ^13^C records suggest a constant dominance of C3 plant species, enabling the interpretation of the δ^13^C_WMA_ record as indicator of plant water stress [71]. We also note, that δ^13^C_WMA_ values are moderately correlated with ACL_29-35_ values (R = 0.442, p = 0.001), further corroborating the use of the δ^13^C_WMA_ record as recorder of past plant water stress in this study. However, δ^13^C_WMA_ values are not significantly correlated to δD_WMA_ values (Table 3) arguing against an influence of plant water stress and further, aridity on the hydrogen isotopic composition within the studied *n*-alkane homologues. Altogether, the ACL_29-35_ and δ^13^C_WMA_ records indicate a minor, if any, influence of aridity and related evapotranspiration on the δD_WMA_ values.

Thus, we assume that these parameters as well as precipitation amount and atmospheric temperature have not been dominant drivers for the observed *n*-alkane hydrogen variability in ODP-161-976A during the analysed period. Another potential driver is the source of precipitation [75]. Indeed, modern analyses of meteorological data from the Western Mediterranean indicate that δD_prec_ values in the area depend on the source of precipitation with Atlantic derived precipitation exhibiting significantly lower δD_prec_ values compared to Mediterranean derived precipitation [48, 86]. Since Atlantic sourced precipitation in the study area is much more prominent during the winter season [30, 48], changes in precipitation source likely account for the apparent correlation of monthly amount-weighted δD_prec_ values with the monthly variability of atmospheric temperature and precipitation amount (Fig 1). *n*-Alkane δD values were also considered to reflect changes in the precipitation source by previous paleoclimatic studies from the Iberian Peninsula [65, 67, 68]. Accordingly, we conclude that the dominant parameter driving hydrogen isotopic variability of individual *n*-alkanes in marine sediment core ODP-161-976A is the source of precipitation with low δD_WMA_ values reflecting increasing Atlantic derived precipitation and high δD_WMA_ values reflecting an increase in Mediterranean sourced precipitation.

### Over-regional driver of climate variability

Based on high δD_WMA_ values we define three major periods of overall enhanced Mediterranean sourced precipitation (Fig 4). These range from ca. 5450 to 5350 cal. BP, from 5150 to 4300 cal. BP including a short-term decrease in δD_WMA_ values around 4800 cal. BP, and from 3400 to 3000 cal. BP with an interruption around 3200 cal. BP. The latter two periods of enhanced Mediterranean sourced precipitation correspond well with times of dominant positive modes of the North Atlantic Oscillation (NAO^+^) [87] and reduced Western Mediterranean storminess [88, 89] (Fig 4). The Western Mediterranean storminess record, however, shows higher variability, which might be related to its distant location in the Gulf of Lions. But generally, NAO^+^ conditions would have favoured a northward shift of the Atlantic storm track towards northern and central Europe [43] (Fig 2). Consequently, this resulted in reduced storminess across the Western Mediterranean as generally evidenced by an increased clay mineral ratio from the Gulf of Lions during dominant positive NAO modes [88]. Along with the northward displacement of the Atlantic storm track, the majority of Atlantic sourced precipitation was shifted to northern and central Europe [90]. This results in high δD_WMA_ values as Atlantic sourced precipitation in southeast Iberia was reduced and the Mediterranean sourced precipitation gained more importance. One exception is observed at ca. 3350 cal. BP, when the NAO reconstruction indicates a prominent change to a negative mode (NAO^-^). These NAO^-^ conditions, however, represent a very abrupt, event-like feature, which might not be recorded in our data.

However, based on the modern NAO-precipitation relationship one would expect an increase in cold season precipitation when the Atlantic influence increases [24, 44]. Therefore, it is interesting to note that the rapid shift in δD_WMA_ values towards relatively increased Atlantic moisture source at 4300 cal. BP is not coincident with elevated cold season precipitation levels (Fig 4). In fact, after 4300 cal. BP cold season precipitation at the south-eastern Iberian Peninsula reveals a prominent decreasing trend. Regarding the 4.2 ka BP event, which occurred during this period, previous studies indicate that this period was characterized by decreasing summer precipitation [6, 7, 28]. This is in line with our reconstruction of regional warm season precipitation, which indicates a rapid decrease between ca. 4200 to 3700 cal. BP (Fig 4). Taken into account that modern warm season precipitation in the area has a dominant Mediterranean source, a significant reduction in warm season precipitation might be able to explain the relative increase in Atlantic sourced precipitation at 4300 cal. BP, even though cold season precipitation was gradually decreasing.

According to the previous finding, a dominant role of the NAO–active during the cold season–as driver for the observed reduction in warm season precipitation during the 4.2 ka BP event is not plausible. However, based on *n*-alkane hydrogen isotopic analysis the Western Mediterranean Oscillation (WeMO) has recently been suggested as potential driver for changes in the precipitation source across southern Iberia during the studied period [68]. During modern times warm season precipitation at the southeast Iberian Peninsula is mainly derived from local convective systems [48], which benefit from a high land-sea temperature contrast (i.e. warm ocean and cool land). Such a high land-sea temperature contrast during the summer months is favoured by a positive mode of the Western Mediterranean Oscillation (WeMO^+^) due to the advection of cool air masses from the north [46, 48] (Fig 2). In contrast, WeMO^-^ conditions would favour a rather low land-sea temperature contrast because warm, easterly winds prevail [46]. Thus, our generally decreased δD_WMA_ data between 4300 and 3400 cal. BP along with the observed reduction in warm season precipitation could have been promoted by persistent WeMO^-^ conditions at that time.

Altogether, the NAO appears to be the dominant modulator of δD_WMA_ values, and thus, relative variability in moisture sources in the studied area between 6000 and 3000 cal. BP. This is plausible because annual precipitation is dominated by cold season precipitation on the Iberian Peninsula, which is also the season when the NAO is active [24]. However, secondary modes active during different seasons such as the WeMO may alter this overall relationship.

## Conclusion

In order to investigate the interaction between Atlantic and Mediterranean climate at the south-eastern Iberian Peninsula during the mid- to late Holocene, compound-specific hydrogen (δD_Cx_) and carbon isotopic records (δ^13^C_Cx_) from fossil leaf waxes (i.e. *n*-alkanes) have been analysed. Detailed comparison with δ^13^C_Cx_ values, sea surface temperature variability, and changes in precipitation amount indicate that δD_Cx_ values and their weighted-mean average (δD_WMA_) analysed in this study are related to changes in the precipitation source. While low δD_WMA_ values are indicative of increased Atlantic origin, high δD_WMA_ values indicate a Mediterranean precipitation source.

Overall, δD_WMA_ variability appears closely related to variability of the North Atlantic Oscillation (NAO) between 6000 and 3000 cal. BP with positive NAO modes resulting in reduced storm activity across the Western Mediterranean and a relative increase of Mediterranean sourced precipitation in southeast Iberia. However, secondary atmospheric modes such as the Western Mediterranean Oscillation (WeMO) may alter this overall relationship. Such an example is the period of the 4.2 ka BP event, which on the southeast of the Iberian Peninsula was identified as a period of a rapid reduction in warm season precipitation between ca. 4200 and 3700 cal. BP. According to low δD_WMA_ values, these reductions in warm season precipitation were related to a decreasing Mediterranean influence during the summer months. While it is not plausible that the NAO serves as driver for the reduced warm season precipitation, a hypothesized influence of the WeMO by lowering the land-sea temperature contrast is in line with a previous study [68].

## Supporting information

S1 Fig Cross-plots and correlations of environmental and paleoclimatological parameters of sediment core ODP-161-976A. (TIF)

S2 Fig Concentration of individual *n*-alkane homologues from sediment core ODP-161-976A. (TIF)
