# Supplementary figures and images for: Fossil leaf wax hydrogen isotopes reveal variability of Atlantic and Mediterranean climate forcing on the southeast Iberian Peninsula between 6000 to 3000 cal. BP
Source: PLoS One. 2020 Dec 23;15(12):e0243662. doi: 10.1371/journal.pone.0243662 (PMC7757796; doi:10.1371/journal.pone.0243662)

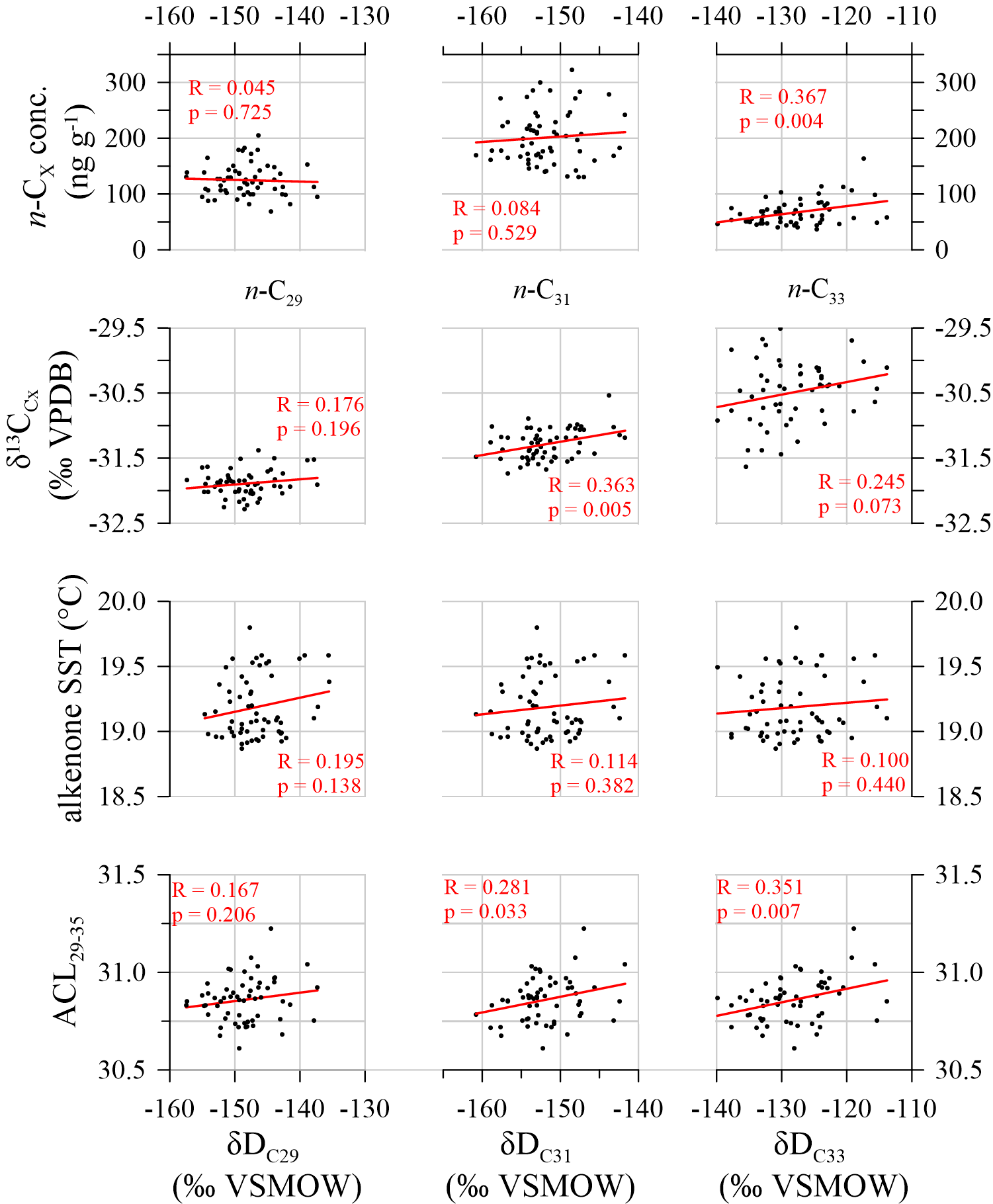

Supplement: S1 Fig — (TIF) [file pone.0243662.s001.tif]

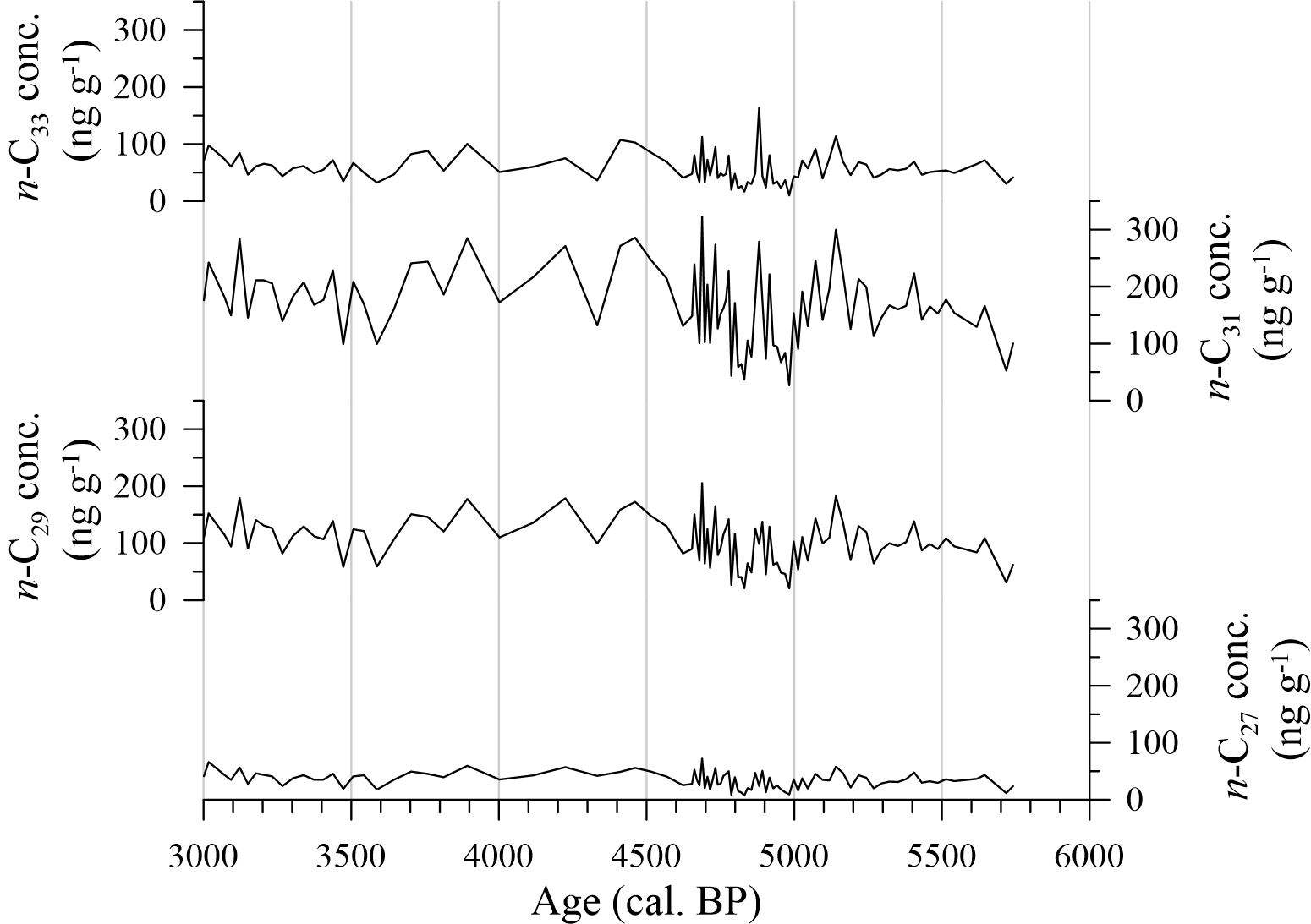

Supplement: S2 Fig — (TIF) [file pone.0243662.s002.tif]
